# Efficacy of switching to bilastine, a histamine H1 receptor antagonist, in patients with chronic spontaneous urticaria (H1-SWITCH): study protocol for a randomized controlled trial

**DOI:** 10.1186/s13063-019-3878-2

**Published:** 2020-01-06

**Authors:** Atsushi Fukunaga, Yoshiko Oda, Ken Washio, Takashi Omori, Yasumasa Kakei, Michihiro Hide, Chikako Nishigori

**Affiliations:** 1 Division of Dermatology, Department of Internal Related, Kobe University Graduate School of Medicine, 7-5-1 Kusunoki-cho, Chuo-ku, Kobe, 650-0017 Japan; 2 Division of Biostatistics, Department of Social/Community Medicine and Health Science, Kobe University Graduate School of Medicine, Kobe, Japan; 3 Department of Oral and Maxillofacial Surgery, Kobe University Graduate School of Medicine, Kobe, Japan; 4 Department of Dermatology, Institute of Biomedical & Health Sciences, Hiroshima University, Hiroshima, Japan

**Keywords:** Chronic spontaneous urticaria, H1-antihistamine, Switching, Bilastine

## Abstract

**Background:**

Chronic spontaneous urticaria (CSU) is characterized by the spontaneous appearance of wheals, angioedema, or both for > 6 weeks. Continuous treatment with H1-antihistamines is used as a first-line treatment for CSU. However, H1-antihistamine treatment leads to absence of symptoms in less than 50% of patients with CSU. Although Japanese guidelines for the diagnosis and treatment of urticaria recommend an increase in the H1-antihistamine dose or a switch to other H1-antihistamines, there is no evidence supporting a switch to other H1-antihistamines in patients with refractory CSU who are unresponsive to H1-antihistamines at the licensed dose.

**Methods:**

We will conduct a multicenter, open-label, non-inferiority, randomized, parallel, comparison study to determine if the efficacy of bilastine 20 mg is not inferior to that of a twofold H1-antihistamine dose increase in patients with refractory CSU who are unresponsive to H1-antihistamines at the licensed dose. This study will be performed at 15 academic hospitals in Japan, and the administration period (increasing the H1-antihistamine dose twofold vs. switching to bilastine 20 mg) will be 7 days. Participants (*n* = 150) will be randomized to either an increased H1-antihistamine dose or a switch to bilastine 20 mg at a 1:1 ratio. The primary endpoint, mean of the total symptom score of 5–7 days after the intervention, will be evaluated. The secondary objective is to determine if the safety of bilastine 20 mg regarding somnolence is superior to that of a twofold dose increase of H1-antihistamines. This will be measured by a change in the Japanese version of the Epworth Sleepiness Scale from baseline to 7 days after starting the intervention.

**Discussion:**

This multicenter, open-label, non-inferiority, randomized, parallel, comparison study will be, to our knowledge, the first well-designed clinical study to evaluate the efficacy of a switch to other H1-antihistamines in patients with refractory CSU who are unresponsive to H1-antihistamines at the licensed doses. This trial will provide evidence of the efficacy and safety of bilastine when treatment is switched in patients with refractory CSU who are unresponsive to H1-antihistamines at the licensed dose.

**Trial registration:**

Japan Registry of Clinical Trials (jRCT), jRCTs051180105. Registered on 8 March 2019.

## Background

Chronic spontaneous urticaria (CSU) is a common skin disease that is characterized by the spontaneous appearance of wheals, angioedema, or both for > 6 weeks as a result of known (i.e., the presence of mast cell-activating autoantibodies) or unknown causes [1, 2]. CSU is highly prevalent, affecting up to 1% of the population, and it has a significant negative impact on a patient’s quality of life and health [3]. Histamine and other mediators, such as platelet-activating factor (PAF) and cytokines released from activated skin mast cells, induce sensory nerve activation, vasodilatation, and plasma extravasation as well as cell recruitment to urticarial lesions.

Although there are several theories about CSU pathogenesis, none have been conclusively established, but it is evident that histamine from activated skin mast cells plays a central role in the pathophysiological aspects of CSU. Many urticaria symptoms are mediated primarily by the actions of histamine on H1 receptors located on endothelial cells (wheal) and on sensory nerves (neurogenic flare and pruritus). CSU is a self-limiting but long-lasting disorder, persisting for 2–5 years in most cases, and 20% of patients are affected for more than 5 years [4]. Therefore, continuous treatment with H1 receptor antagonists (H1-antihistamines) is important when treating patients with CSU. Modern second-generation antihistamines should be considered to be the first-line symptomatic treatment for CSU because of their good safety profile [1].

However, H1-antihistamine treatment leads to the absence of symptoms in less than 50% of patients with CSU [5]. In refractory CSU patients who are unresponsive to H1-antihistamines at the licensed dose, the benefits of a higher dosage of second-generation antihistamines have been shown [6–8]. Therefore, the European Academy of Allergy and Clinical Immunology (EAACI)/Global Allergy and Asthma European Network (GALEN)/European Dermatology Forum (EDF)/World Allergy Organization (WAO) guidelines recommend that doses of second-generation H1-antihistamines should be increased up to fourfold higher in patients with chronic urticaria that is unresponsive to the licensed dose of second-generation H1-antihistamines [1]. However, Japanese guidelines for the diagnosis and treatment of urticaria recommend either switching to other H1-antihistamines, combined use, or increasing the dose in patients who are unresponsive to second-generation H1-antihistamines at the licensed dose [9]. However, no study has provided high-quality evidence about the efficacy of switching to other H1-antihistamines in patients with CSU that is resistant to a certain H1-antihistamine.

O’Donnell et al. showed that health status scores in patients with CSU are comparable to those reported by patients with coronary artery disease [10]. Therefore, the goal of CSU treatment is to treat the disease until it is gone. However, the average age of CSU patients ranges mostly from adolescence to middle age, i.e., productive ages, and symptomatic relief and an improvement in labor productivity without compromising the patient’s quality of life are important. Because H1-antihistamine is an essential first-line treatment for CSU, and somnolence is one of its well-known side effects, it is important that clinicians choose a treatment that does not impair labor productivity and quality of life.

Bilastine, a non-sedating second-generation H1-antihistamine, has been approved in 90 countries for therapeutic use in patients with urticaria and allergic rhinitis, with a recommended dose of 20 mg once daily in patients older than 12 years. A clinical pharmacological study using positron emission tomography demonstrated that a single oral dose of bilastine 20 mg did not occupy H1 receptors in the brain [11]. The total symptom score (TSS), which is defined as the sum of the scores for rashes and itching, was significantly improved at the early stage (Days 1–3) in the group given bilastine 20 mg once daily compared with placebo [12]. Moreover, long-term treatment with bilastine 20 mg once daily for 52 weeks has been shown to be safe and well tolerated in Japanese patients with CSU [13]. The study reported that somnolence related to bilastine was reported in only two of 197 patients with CSU (1.0%), a considerably lower value than that reported in other second-generation H1-antihistamine clinical studies.

### Objectives

#### Primary objectives

The primary objective is to determine if the efficacy of bilastine 20 mg (licensed dose) is not inferior to that of a twofold increase in the dose of H1-antihistamines in subjects with CSU who are resistant to H1-antihistamine treatment at the licensed doses.

#### Secondary objectives

The key secondary objective is to determine if the safety of bilastine 20 mg regarding somnolence is superior to a twofold increase in the H1-antihistamine dose in subjects with H1-antihistamine-resistant CSU based on H1-antihistamine treatment at licensed doses.

## Methods/design

### Study design

This study is designed as a multicenter, open-label, non-inferiority, randomized, parallel, comparison study.

### Study setting

This study will be performed at 15 academic hospitals in Japan. All study data will be stored and archived in the data center at Kobe University Hospital.

### Study population

#### Inclusion criteria

The inclusion criteria are as follows:

1. Patients aged 20 years or older at the time that they provide consent
2. Patients diagnosed with CSU, which is characterized by the spontaneous appearance of wheals, angioedema, or both for > 6 weeks without any triggers, for which second-generation non-sedative or mild-sedative antihistamines (regular dose) is not sufficiently effective at the time of randomization
3. Patients with CSU with pruritus or wheal continuation despite continued treatment with second-generation non-sedating or mild-sedative antihistamines (regular dose) for more than 1 week before consent acquisition. However, it is permissible to change the type of antihistamine within the range of dosage and usage information on the medication information leaflet. Second-generation non-sedating antihistamines include fexofenadine hydrochloride, levocetirizine, olopatadine hydrochloride, bepotastine besilate, loratadine, cetirizine hydrochloride, epinastine hydrochloride, ebastine, lupantadine fumarate, azelastine hydrochloride, and mequitazine
4. Patients with an Urticaria Control Test (UCT) score of 11 or less on the registration date (The UCT can evaluate retrospectively the level of urticaria control over the past 4 weeks using four questionnaires with a recommended cutoff value of 12 for controlled disease.)
5. Patients for whom documented consent has been obtained regarding their voluntary participation in this clinical study
6. Patients who are able to take the test drug or control drug for 7 days even if symptoms (pruritus or wheal) improve within this period.

#### Exclusion criteria

The exclusion criteria are as follows:

1. Patients with urticaria, other than CSU, with an identifiable trigger/cause. If triggering factors are specified so that the symptoms developed in response to this factor can be clearly distinguished from those of CSU, they are not considered to conflict with the exclusion criteria
2. Patients with a skin disease accompanied by chronic pruritus other than CSU (eczema, contact dermatitis, and atopic dermatitis)
3. Patients with hypersensitivity to bilastine
4. Patients with chronic, uncontrolled medical condition(s) that may increase the risk of study subjects by participating in this clinical study, based on the study investigator’s or study team physician’s judgment
5. Pregnant or lactating women
6. Patients treated with bilastine, adrenocorticosteroid, or cyclosporine within 4 weeks before obtaining consent. Patients treated with first-generation antihistamines, H2 receptor antagonists, or antileukotriene drugs within 1 week before obtaining consent. However, any use of external medicines is permitted
7. Patients with CSU treated with omalizumab in the past
8. Patients who are judged as inappropriate by a study investigator or sub-investigators.

### Intervention

There will be two groups studied:

1. Group A, increasing the H1-antihistamine dose to twofold higher than the regular dose. H1-antihistamine dose (regular dose) administered orally before registration will be increased twofold and orally administered. The number of oral doses during the day will not change. The administration period will be 7 days, and the H1-antihistamine will be taken daily beginning on Day 1 (first prescription day). Medications administered twice daily will be taken until the morning of Day 8.
2. Group B, switched to the regimen of bilastine 20 mg. Bilastine is orally administered once daily at least 1 h before dinner. The administration period is 7 days, and bilastine will be taken daily beginning on Day 1 (first prescription day).

### Randomization (allocation)

Subjects will be randomly assigned to either the bilastine group or the twofold H1-antihistamine group at a 1:1 allocation, using the permutation random block method stratified by UCT category (less than 8 points, or greater than or equal to 8 points). The block sizes will not be disclosed to ensure that blinding is maintained. The allocation sequence for the randomization method will be generated by the biostatistician.

All the subjects who provide consent to participate and who fulfill the inclusion criteria and do not meet any of the exclusion criteria will be randomized. The principal investigator or sub-investigator will send a Subject Enrollment Form by Fax to the data center. The staff at the data center will confirm the subject’s eligibility and issue the Subject Enrollment Confirmation Form, which contains the eligibility judgment result, the randomization assignment result from the generated random sequence, and the enrollment number. Thereafter, the form will be sent to the principal investigator or sub-investigator.

### Outcomes

The EAACI/GALEN/EDF/WAO guidelines and Japanese guidelines for Diagnosis and Treatment of Urticaria 2018 recommend using the Urticaria Activity Score (UAS) [7] and the UCT to assess the activity and control of CSU [1]. However, the TSS, which is defined as the sum of the rash and itch scores, will be used as the primary endpoint for efficacy in this open-label, multicenter, phase III study to evaluate the long-term safety and efficacy of bilastine, a novel non-sedating H1-antihistamine, for Japanese patients with CSU [13]. Therefore, we also set the TSS as the primary endpoint in this study. As quality of life measures, the Japanese version of the Epworth Sleepiness Scale (JESS) will be used to evaluate safety including somnolence parameters, and the Dermatology Life Quality Index (DLQI) to measure the impact of CSU.

### Primary endpoint

The primary endpoint is the mean of the TSS 5–7 days after intervention.

### Secondary efficacy endpoints

The secondary efficacy endpoints are the following:

- UAS7 (the sum of the daily UAS scores over 7 consecutive days)
- Change from baseline in the total DLQI score at 7 days after starting the intervention
- Change in the average TSS from 3 days before the intervention to the average TSS at Days 5–7 after starting the intervention

### Secondary endpoints for safety

The endpoints for safety are the:

- Change in JESS from baseline to 7 days after starting the intervention (important secondary endpoint)
- Presence or absence of disease that is associated with the conduct of this clinical study

### Time schedule of intervention, outcomes, and other assessments

The relationship of intervention, outcomes, other assessments, and visits for subjects in this study is shown in Table 1.

**Table 1:**
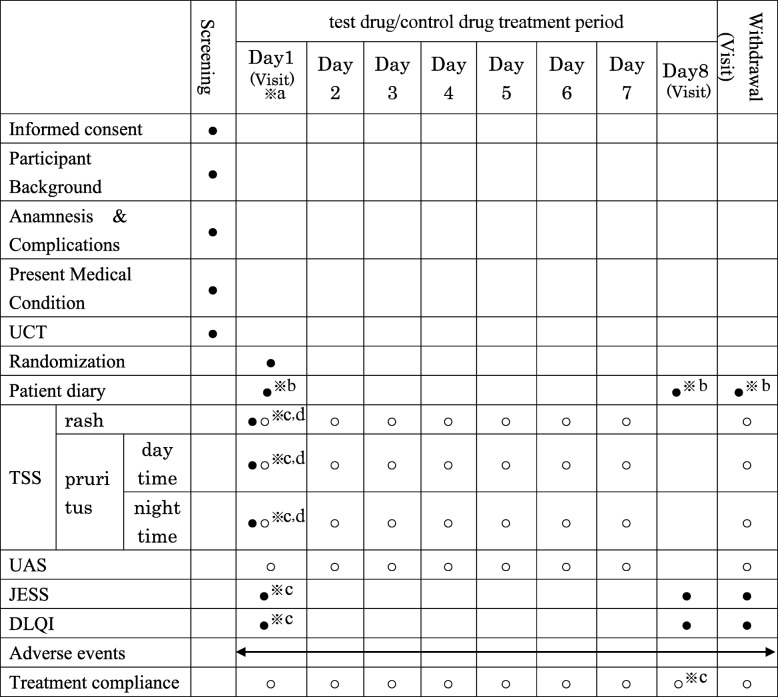
Summary of study assessments and procedures

● Performed by a physician at a medical institution

○ Study subjects to perform at home

^a^Screening and Day 1 may be performed on the same day

^b^Patient diary is delivered on Day 1 and collected on Day 8 or upon withdrawal from the study

^c^TSS, JESS, and DLQI will be administered before taking the test drug or control drug on Day 1

^d^For TSS, the situation in the past 3 days before the start of treatment will be investigated

^e^The morning oral condition will be confirmed on Day 8

### Data collection and management

The primary investigator or sub-investigator will enter the case report form (CRF) data for each subject into the electronic data capture (EDC) system. The principal investigator will confirm that the entered CRF data is complete and correct, electronically sign the CRF on the EDC system, and then make a printout of the signed CRF for filing. The CRF printout will be retained. If there are any queries about the CRF data that are entered by the staff at the data center, the primary investigator or sub-investigator should promptly respond to the queries.

### Statistics: sample size calculation

The target number of subjects is 150, with 75 subjects in group A and 75 subjects in group B.

We have designed the trial for the non-inferiority of bilastine compared with other H1-antihistamines for TSS and the superiority of bilastine compared with other H1-antihistamines for JESS. For the superiority, we do not set any margin. As there should be just one primary endpoint, we positioned the TSS as the primary endpoint and the JESS as the important secondary endpoint. The sample size was determined based on the primary endpoint. To confirm the superiority for the important secondary endpoint, the statistical testing for JESS will be limited to only when the non-inferiority for the primary endpoint, TSS, is shown. This will allow us to avoid type I error inflation.

According to the domestic phase III randomized controlled trial that was conducted to obtain approval to use bilastine to treat CSU, the mean ± standard deviation of the change from baseline to 1 week in the 20 mg/day group, as assessed using the TSS, was − 2.75 ± 1.55, while that in the placebo group was − 1.05 ± 1.01 [13, 14]. Therefore, the difference in means between both groups is about 1.7. Based on this value, the non-inferiority margin was set as 0.8, which is less than half of the difference in the two means. Also, the mean ± standard deviation of the TSS values 1 week after administration of bilastine, 20 mg/day in the phase III study, was 1.79 ± 1.27. Because our trial is a drug switching study (which is different from the reference trial), the standard deviation may be larger. Therefore, the common standard deviation of the mean TSS values ​5–7 days after bilastine administration in group B and in group A was estimated to be 1.7. The difference between the two means was assumed to be 0.

Under the preceding assumptions, and using a non-inferiority margin of 0.8, with a one-sided significance level of 2.5% and a statistical power of 80%, the number of needed subjects based on a statistical test to confirm non-inferiority of bilastine compared with other H1-antihistamines was estimated to be 71 per group. Considering the uncertainty and omissions that result from estimation, the sample size was set to 75 subjects per group. With 71 subjects per group, the detection power is 75%, 70%, and 66% with a standard deviation of 1.8, 1.9, and 2.0, respectively.

Saruwatari et al. [15] reported results using the JESS to assess adverse events of H1-antihistamines. From these results, we can estimate that the difference of means between the H1-antihistamines group and the control group is around 1 and the standard deviation is around 5.5. Even if the difference of means is set as 0.5, under a two-sided significance level of 5% and a detection level of 80%, the needed number of subjects is 65 per group. Therefore, using 75 subjects per group as the needed number of subjects in this study has enough power to detect the important secondary endpoint.

### Analysis

A summary of the planned statistical analysis for this study is provided in the following discussion. Details of the planned statistical analysis will be described in the Statistical Analysis Plan. The final analysis will be performed after data from the subjects have been obtained and locked after the end of the follow-up period.

The analysis populations for efficacy assessment are the full analysis set (FAS) and the per protocol set (PPS). We will analyze data from these two populations for the purpose of sensitivity analyses. The primary analyses will be conducted using the FAS. The FAS is defined to consist of all subjects enrolled in this study who were administered at least one dose of the study drug after randomization, excluding absence of informed consent and enrollment outside the contract period. The PPS is defined to consist of the subjects in the FAS, excluding those with any of the following significant protocol violations such as, but not limited to, those involving the study method or concomitant therapy:

1. No baseline data
2. Violation of the inclusion criteria
3. Violation of the exclusion criteria
4. Violation of prohibited concomitant medications
5. Violation of prohibited concomitant treatments
6. Violation of compliance (less than 80%).

The analysis population for the safety assessment is the safety analysis set, which will consist of the subjects enrolled in this study who were given at least one dose of the study drug or control drug.

If there is any doubt about the data summarization or analysis, the biostatistician and the study representative will discuss the issue and decide how to handle it. In principle, missing values will be imputed as necessary. Details will be described in the Statistical Analysis Plan.

### Primary analysis

#### Primary endpoint

The primary endpoint, the mean TSS after 5–7 days, will be analyzed using analysis of covariance (ANCOVA) including two factors: UCT category and treatment group. A statistical test for the adjusted difference in the means of the primary endpoint between both groups with ANCOVA for a non-inferiority margin of 0.8 will be performed using a significance level of 0.025. The adjusted difference in means of the primary endpoint between both groups and its 95% confidence interval will also be estimated. If a TSS value during days 5–7 is missing, the value of day 4 will be imputed and the mean TSS will be calculated. If more than two TSS values during days 5–7 days are missing, the data will be eliminated in the analysis of the primary endpoint.

#### Important secondary endpoint

In order to confirm the superiority of group B compared to group A, and only if the primary analysis for the primary endpoint shows a statistical significance, a one-sided test for the mean JESS value at 7 days using ANCOVA including two factors, UCT category and treatment group, with a significance level of 0.025 will be performed. The adjusted difference in means between both groups with the ANCOVA and its 95% confidence interval will also be estimated.

### Secondary analysis

Three secondary endpoints, UAS7, change in DLQI at 7 days from baseline, and change in the mean TSS 5–7 days after starting treatment compared with the mean 3 days before treatment administration, will be analyzed using ANCOVA including the two factors of UCT category and treatment group. The adjusted difference in means between both groups for these secondary endpoints and its 95% confidence interval will be estimated, and statistical tests for the adjusted difference will also be performed at a significance level of 0.025.

### Adverse events

In our study, an adverse event is defined as any disease, disability, death, or infection that occurs during this study. The principal investigator or sub-investigator will record all adverse events in the CRF and treat and follow the patient(s) until resolution during the study. If the principal investigator or sub-investigator finds a potentially causal relationship to the study drug, all adverse events will be recorded to report to the review board.

### Monitoring and auditing

Periodic monitoring of the study will be performed to check that the human rights and welfare of subjects are being protected, the study is being conducted safely in accordance with the protocol and the applicable regulatory requirements under the Clinical Trials Act, and the data are being collected properly. The principal investigator will appoint a responsible monitor and other monitors as needed for the study. The items to be checked at monitoring are specified in the “Written procedure for implementation of study monitoring.”

For quality assurance, the study will be examined to determine that it is being conducted in accordance with the protocol and written procedures, independently and separately from the routine activities of monitoring and quality control. The study representative will complete the “Written procedure auditing” form and will ensure that the appointed auditor audits the study in accordance with the “Written procedure for auditing.” The auditor will report the audit results to the study representative and the principal investigator at the site that was selected for the audit.

## Discussion

CSU is a common skin disease, and it is evident that histamine from activated skin mast cells plays a central role in its pathophysiological aspects. The symptomatic pharmacological treatment aims for complete CSU symptom relief. Continuous treatment with second-generation H1-antihistamines is recommended as the first-line treatment for CSU. However, to date, there are few well-designed clinical studies comparing the efficacy and safety of different modern second-generation H1-antihistamines in urticaria [1]. Additionally, H1-antihistamine treatment leads to the absence of symptoms in less than 50% of patients with CSU [5], and only a few studies showed the benefit of a higher dosage of a second-generation antihistamine for patients with CSU who were unresponsive to the licensed dose H1-antihistamines. The Japanese guidelines for diagnosis and treatment of urticaria recommend switching to another H1-antihistamine, combined use, or increasing the dose in patients who are unresponsive to a second-generation H1-antihistamine at the licensed doses [9]. Switching to another H1-antihistamine is a common medical practice in patients with refractory CSU. However, no studies with high-quality evidence have reported the efficacy of switching to another H1-antihistamine in patients with CSU resistant to H1-antihistamine treatment. This study will provide evidence for the efficacy of bilastine, a modern non-sedative H1-antihistamine, on the switching treatment for patients with CSU who are unresponsive to second-generation H1-antihistamines at the licensed doses.

The EAACI/GALEN/EDF/WAO guideline recommends the use of second-generation H1-antihistamines at up to a fourfold higher dose in patients with CSU who are unresponsive to second-generation H1-antihistamines at the standard dose. In this study, a twofold increase in the dose of second-generation H1-antihistamines was chosen, because an increase beyond twice the recommended dose is not permitted under the medical insurance system in Japan. Therefore, the protocol of this study reflects real-world medical practice for patients with CSU in Japan.

The brain penetration of orally administered cetirizine, another second-generation H1-antihistamine, was found to be dose-dependent [16]. This report concluded that cetirizine 10 mg, a second-generation H1-antihistamine with a low H1 receptor occupancy in the central nervous system, could be more safely used for the treatment of allergic disorders, while an increased dose (20 mg or more) could result in mild sedation. Other second-generation H1-antihistamines also tend to cause dose-related cognitive impairment in certain patients at higher doses. Therefore, somnolence and the strength of sedation will be assessed using the JESS as a secondary endpoint in this study, to evaluate the improvement in labor productivity without compromising the patient’s quality of life. Quality of life is also evaluated using the DLQI as another secondary endpoint in this study to check whether quality of life is impaired following a change in medication. It would be useful in daily clinical practice to examine the influence of this change in treatment on other health problems, as well as the effectiveness of treatment after the change in patients with CSU.

Bilastine, a novel non-sedating second-generation H1-antihistamine, has been widely used since 2010 to treat allergic rhinoconjunctivitis and urticaria with a recommended dose of 20 mg once daily. Bilastine requires no dose adjustment for patients with renal impairment [17]. It has also been reported that a single oral dose of bilastine 20 mg had minimal H1 histamine receptor occupancy in the brain, based on positron emission tomography criteria [12]. Additionally, a study found that the TSS was significantly improved in the early stage (Days 1–3) in the bilastine 20 mg once daily group in Japanese patients with CSU, confirming the rapid efficacy of bilastine for these patients [13]. Similar to bilastine 20 mg, bilastine 10 mg, which is lower than the licensed dose in Japan, significantly decreased TSS compared to the placebo [13]. Because bilastine was approved as a single dose of 20 mg, higher doses are not permitted under medical insurance in Japan. Therefore, we chose bilastine as the test drug to switch to from other H1-antihistamines that were not effective in treating patients with CSU, and we decided that a 1-week study period for treatment was a sufficient short-term period. The UCT, which is part of the inclusion criteria, is a retrospective assessment tool for the activity and control of CSU, and it has been used to objectively evaluate patients with refractory disease [18, 19]. Adverse events and all diseases that occur will be recorded and observed until the disease resolves within the study period, regardless of a causal relationship with the clinical study.

### Trial status

The study was first authorized on February 4, 2019. Participant recruitment will start on June 10, 2019. The expected date of completion (last visit of last patient) is the end of December 2020. Central ethical approval has been confirmed from the Kobe University Clinical Research Ethics Committee (reference approval number C180046), and we will not begin recruiting at other centers in the trial until local ethical approval has been obtained. The Standard Protocol Items: Recommendations for Interventional Trials (SPIRIT) checklist is provided as Additional file 1, Fig. 1.

**Fig. 1:**
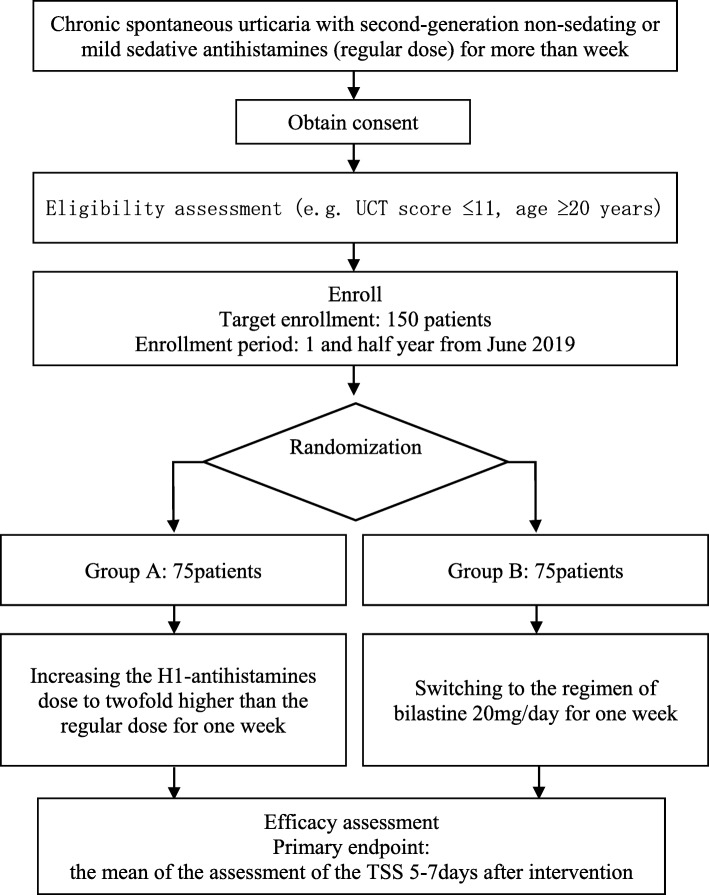
A flowchart of the study design

## Supplementary information

**Additional file 1.** SPIRIT 2013 checklist: recommended items to address in a clinical trial protocol and related documents.

## Data Availability

Not applicable.

## Abbreviations

ANCOVA: Analysis of variance
CRF: Case report form
CSU: Chronic spontaneous urticaria
DLQI: Dermatology Life Quality Index
EAACI: European Academy of Allergy and Clinical Immunology
EDC: Electronic data capture
EDF: European Dermatology Forum
FAS: Full analysis set
GALEN: Global Allergy and Asthma European Network
JESS: Japanese version of Epworth Sleepiness Scale
PPS: Per protocol set
SD: Standard deviation
TSS: Total symptom score
UAS: Urticaria Activity Score
UCT: Urticaria Control Test
WAO: World Allergy Organization
